# Supplementary material for: Spatio-Temporal Variation of Conversational Utterances on Twitter
Source: PLoS One. 2013 Oct 31;8(10):e77793. doi: 10.1371/journal.pone.0077793 (PMC3814942; doi:10.1371/journal.pone.0077793)
Supplement: Text S1 — Information on State and County QuickFacts variables. (PDF) [file pone.0077793.s003.pdf]

Supporting information: *Spatio-temporal variation of conversational utterances on Twitter*

Christian M. Alis, May T. Lim

September 30, 2013

1 List of State and County Quick Facts variables

1. Resident total population estimate (July 1) 2011
2. Resident total population, estimates base (April 1) 2010
3. Resident total population, percent change - April 1, 2010 to July 1, 2011
4. Resident population (April 1 - complete count) 2010
5. Resident population under 5 years, percent, 2011
6. Resident population under 18 years, percent, 2011
7. Resident population 65 years and over, percent, 2011
8. Resident population: total females, percent, 2011
9. Resident population: White alone, percent, 2011
10. Resident population: Black alone, percent, 2011
11. Resident population: American Indian and Alaska Native alone, percent, 2011
12. Resident population: Asian alone, percent, 2011
13. Resident population: Native Hawaiian and Other Pacific Islander alone, percent, 2011
14. Resident population: Two or more races, percent, 2011
15. Resident population: Hispanic or Latino Origin, percent, 2011
16. Resident population: Not Hispanic, White alone, percent, 2011
17. Population 1 year and over by residence - same house, one year ago, percent, 2007-2011
18. Place of birth, foreign born, percent, 2007-2011
19. Population 5 years and over, percent speaking language other than English at home, 2007-2011
20. Educational attainment - persons 25 years and over - percent high school graduate or higher, 2007-2011
21. Educational attainment - persons 25 years and over - percent bachelor's degree or higher, 2007-2011
22. Veterans - total, 2007-2011
23. Average travel time to work for workers 16 years and over not working at home, 2007-2011
24. Housing unit estimates, 2011
25. Owner-occupied housing units - percent of total occupied housing units, 2007-2011
26. Housing units by units in structure - multi-dwelling structure, percent, 2007-2011

27. Median value of specified owner-occupied housing units, 2007-2011
28. Households, 2007-2011
29. Average household size, 2007-2011
30. Per capita income in the past 12 months (in 2011 inflation-adjusted dollars), 2007-2011
31. Median household income, 2007-2011
32. People of all ages in poverty, percent, 2007-2011
33. Private nonfarm establishments, 2010
34. Private nonfarm employment for pay period including March 12, 2010
35. Private nonfarm employment for pay period including March 12, 2010, percent change, 2000-2010
36. Nonemployer: total (NAICS 00) - establishments, 2010
37. Total number of firms, 2007
38. Total Black-owned firms, percent, 2007
39. Total American Indian- and Alaska Native-owned firms, percent, 2007
40. Total Asian-owned firms, percent, 2007
41. Total Native Hawaiian- and Other Pacific Islander-owned firms, percent, 2007
42. Total Hispanic-owned firms, percent, 2007
43. Total Women-owned firms, percent, 2007
44. Manufacturing: total (NAICS 31-33) - value of shipments, 2007
45. Wholesale trade: merchant wholesalers (NAICS 42) - sales of establishments with payroll, 2007
46. Retail trade: total (NAICS 44-45) - sales of establishments with payroll, 2007
47. Retail trade: total (NAICS 44-45) - sales of establishments with payroll per capita, 2007
48. Accommodation and Food Services: total (NAICS 72) - sales of establishments with payroll, 2007
49. New private housing units authorized by building permits - total, 2011 (20,000-place universe)
50. Land area in square miles, 2010
51. Population per square mile, 2010

2 Detailed description of variables

2.1 Percent Black resident population in 2011

The percent Black resident population in 2011 is officially described as *Resident population: Black alone, percent, 2011* and comes from the US Bureau of the Census, Population Estimates Program [1]. The race data used in the estimate is based on answers to the 2010 US Census form question, "What is Person X's race?" where X is a number starting from 1 (Fig. S1). Below the question are several choices but a race that is not in the choices may also be specified. A person can have multiple races (2.3% of the entire US population in 2011) but only those that selected a single race may be counted in this variable: hence the word *alone* in the official description. The form does not specify the description of each race and thus answers are entirely based on self-identification. People counted in this variable are those who indicated their race as "Black, African Am., or Negro" or reported entries such as African American, Kenyan, Nigerian, or Haitian.

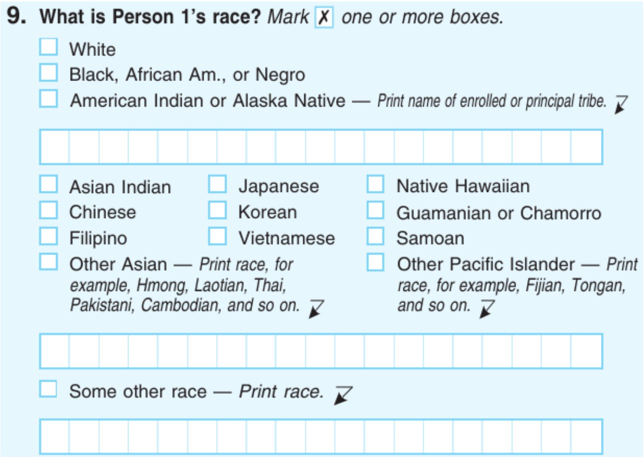

9. What is Person 1's race? *Mark* ☒ *one or more boxes.*

- ☐ White
- ☐ Black, African Am., or Negro
- ☐ American Indian or Alaska Native — *Print name of enrolled or principal tribe.*
- ☐ Asian Indian
- ☐ Chinese
- ☐ Filipino
- ☐ Other Asian — *Print race, for example, Hmong, Laotian, Thai, Pakistani, Cambodian, and so on.*
- ☐ Japanese
- ☐ Korean
- ☐ Vietnamese
- ☐ Native Hawaiian
- ☐ Guamanian or Chamorro
- ☐ Samoan
- ☐ Other Pacific Islander — *Print race, for example, Fijian, Tongan, and so on.*
- ☐ Some other race — *Print race.*

Figure S1: Race information question in the 2010 US Census form

2.2 Percent Black-owned firms in 2007

The percent Black-owned firms in 2007 is officially described as *Total Black-owned firms, percent, 2007* and comes from the US Bureau of the Census, 2007 Economic Census: Survey of Business Owners [2]. A firm is defined as a business that may own several establishments or physical locations of business. An owner is defined as "having 51 percent or more of the stock or equity in the business" [2]. Not included in the Black-owned firms category are publicly held firms, foreign-owned companies, and not-for-profit companies but are in included in the denominator for the total number of firms. The race data is based on the question, "What is Owner X's race?" where X is a number starting from 1 (Fig. S2). Similar to the census, a person may select multiple races and are entirely based on self-identification. The firm is counted as belonging to the category of each selected race.

2.3 Percent of persons 25 years and over who are high school graduates or higher from 2007 to 2011

The percent of persons 25 years and over who are high school graduates or higher from 2007 to 2011 is officially described as *Educational attainment - persons 25 years and over - percent high school graduate or higher, 2007-2011* and comes from the US Bureau of the Census, American Community Survey, 5-Year Estimates [3]. It is an estimate over the period 2007–2011 and based on aggregated yearly answer to the question, "What is the highest degree or level of school this person has COMPLETED?" (Fig. S3). The percentage is obtained by dividing the counts of graduates by the total number of persons 25 years old and over.

2.4 Median household income from 2007 to 2011

The median household income from 2007 to 2011 is officially described as *Median household income, 2007-2011* and comes from the US Bureau of the Census,

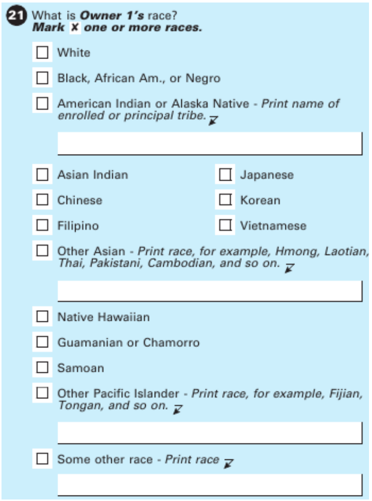

21 What is ***Owner 1's*** race?
Mark X one or more races.

- ☐ White
- ☐ Black, African Am., or Negro
- ☐ American Indian or Alaska Native - *Print name of enrolled or principal tribe.*
- ☐ Asian Indian
- ☐ Chinese
- ☐ Filipino
- ☐ Japanese
- ☐ Korean
- ☐ Vietnamese
- ☐ Other Asian - *Print race, for example, Hmong, Laotian, Thai, Pakistani, Cambodian, and so on.*
- ☐ Native Hawaiian
- ☐ Guamanian or Chamorro
- ☐ Samoan
- ☐ Other Pacific Islander - *Print race, for example, Fijian, Tongan, and so on.*
- ☐ Some other race - *Print race*

Figure S2: Race information question in the 2007 Survey of Business Owners form

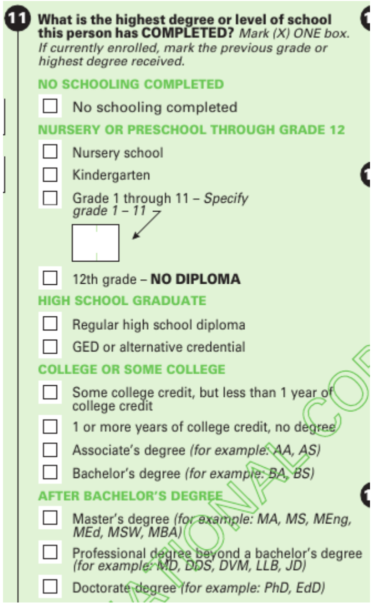

11 **What is the highest degree or level of school this person has COMPLETED?** *Mark (X) ONE box.*
If currently enrolled, mark the previous grade or highest degree received.

NO SCHOOLING COMPLETED

- ☐ No schooling completed

NURSERY OR PRESCHOOL THROUGH GRADE 12

- ☐ Nursery school
- ☐ Kindergarten
- ☐ Grade 1 through 11 – *Specify grade 1 – 11*
- ☐ 12th grade – **NO DIPLOMA**

HIGH SCHOOL GRADUATE

- ☐ Regular high school diploma
- ☐ GED or alternative credential

COLLEGE OR SOME COLLEGE

- ☐ Some college credit, but less than 1 year of college credit
- ☐ 1 or more years of college credit, no degree
- ☐ Associate's degree *(for example: AA, AS)*
- ☐ Bachelor's degree *(for example: BA, BS)*

AFTER BACHELOR'S DEGREE

- ☐ Master's degree *(for example: MA, MS, MEng, MEd, MSW, MBA)*
- ☐ Professional degree beyond a bachelor's degree *(for example: MD, DDS, DVM, LLB, JD)*
- ☐ Doctorate degree *(for example: PhD, EdD)*

Figure S3: Education attainment question in the American Community Survey form

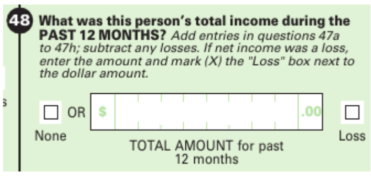

Figure S4: Education attainment question in the American Community Survey form

American Community Survey, 5-Year Estimates [4]. It is an estimate over the period 2007–2011 and based on aggregated yearly answer to the question, "What was this person's total income during the PAST 12 Months?" (Fig. S4). The household income include the income of all members of the household who are at least 15 years old whether they are related to the householder or not. Only members of the household at the time of interview is included.

References

[1] US Census Bureau. Information on race. http://quickfacts.census.gov/qfd/meta/long_RHI225211.htm. URL `http://quickfacts.census.gov/qfd/meta/long_RHI225211.htm`.

[2] US Census Bureau (2012). Survey of business owners - about the survey. http://www.census.gov/econ/sbo/about.html. URL `http://www.census.gov/econ/sbo/about.html`.

[3] US Census Bureau. High school graduates. http://quickfacts.census.gov/qfd/meta/long_EDU635211.htm. URL `http://quickfacts.census.gov/qfd/meta/long_EDU635211.htm`.

[4] US Census Bureau. Median household income. http://quickfacts.census.gov/qfd/meta/long_INC110211.htm. URL `http://quickfacts.census.gov/qfd/meta/long_INC110211.htm`.
